# Determinants of Mobile Learning Behavioral Intention Usage Among Nurses: A Nexus of UTAUT2 Model and Information System Success Model

**DOI:** 10.1155/jonm/5429067

**Published:** 2026-09-23

**Authors:** Tsui Fen Chang, Cheng Min Chao, Wei Ru Chen

**Affiliations:** ^1^ Nursing Department, Yunlin Christian Hospital, Xiluo, Taiwan; ^2^ Department of Nursing, Meiho University, Neipu, Taiwan, meiho.edu.tw; ^3^ Department of Business Administration, National Taichung University of Science and Technology, Taichung, Taiwan, nutc.edu.tw; ^4^ Department of Industrial Engineering and Management, Chaoyang University of Technology, Taichung, Taiwan, cyut.edu.tw

**Keywords:** anxiety, educational technology, mobile applications, nursing, self-efficacy, technology acceptance

## Abstract

**Background:**

Mobile learning provides nurses with a flexible and convenient approach to continuing education and professional development. As nurses are required to continuously update their knowledge and skills, understanding the factors influencing their intention to adopt mobile learning is important for improving nursing education and management practices.

**Methods:**

This study developed an integrated research model based on the Extended Unified Theory of Acceptance and Use of Technology (UTAUT2), the Information System Success Model, and individual psychological factors, including mobile self‐efficacy and technology anxiety. Data were collected from 173 nurses. Structural equation modeling was used to examine the determinants of nurses’ behavioral intention to adopt mobile learning.

**Results:**

The findings showed that attitude, facilitating conditions, and habit were significant positive determinants of behavioral intention, whereas effort expectancy had a negative effect on behavioral intention. Performance expectancy and mobile self‐efficacy were significant predictors of attitude. Effort expectancy and information quality had significant positive effects on performance expectancy, whereas mobile self‐efficacy and technology anxiety had negative effects on performance expectancy. In addition, system quality was identified as a key determinant of effort expectancy.

**Conclusion:**

The results indicate that nurses’ intention to adopt mobile learning is influenced by technological, psychological, and system‐related factors. In particular, attitude, facilitating conditions, habit, performance expectancy, information quality, and system quality play important roles in shaping acceptance of mobile learning.

**Implications for Nursing Management:**

Healthcare administrators and nursing managers should improve system quality, provide sufficient technical support, and strengthen nurses’ confidence and positive attitudes toward mobile learning. These strategies may enhance nurses’ acceptance of mobile learning and support more effective continuing nursing education.

## 1. Introduction

Continuing nursing education (CNE) and continuing professional development (CPD) are essential for practicing nurses to maintain and update the knowledge, skills, and competencies required for high‐quality and safe patient care. Rapid advances in healthcare knowledge, digital technologies, and clinical practices require nurses to engage in continuous learning throughout their professional careers. In particular, the ongoing digital transformation of healthcare has increased the need for nurses to develop and continuously update their digital competencies. However, recent evidence suggests that structured continuing education programs specifically designed to develop digital competencies among nursing professionals remain limited [1].

Mobile learning (M‐learning) provides a flexible approach to CNE and CPD by enabling practicing nurses to access educational resources through smartphones and other mobile devices regardless of time and location. Such flexibility is particularly relevant to nurses, whose participation in conventional continuing education may be constrained by shift work, clinical responsibilities, and limited learning time. Recent evidence also supports the applicability of M‐learning in CNE and professional development. For example, Lee and Chang [2] examined 212 intensive care nurses in northern Taiwan and found that perceived usefulness (PU) was an important factor influencing nurses’ acceptance of M‐learning, while perceived ease of use (PEOU) strongly influenced PU. These findings suggest that the potential benefits of M‐learning depend not only on its availability but also on nurses’ perceptions and acceptance of technology.

Despite the growing use of mobile technologies in nursing practice, their professional application and acceptance remain variable. In a 2024 survey of Australian nurses, 68.4% reported personally using at least one mobile health (mHealth) application, and 58.9% used mHealth applications at least several times per month to access clinical guidelines, protocols, or reference resources [3]. The adoption of emerging AI‐supported technologies is also becoming evident among practicing nurses. A recent study involving 330 registered nurses in Taiwan reported that 46.7% had used ChatGPT, indicating nurses’ increasing exposure to AI‐supported digital tools in the context of self‐directed and professional learning [4]. Taken together, these findings highlight the growing relevance of M‐learning to CNE and professional development among practicing nurses. However, suboptimal adoption of M‐learning may result in underutilization of digital continuing education resources, limiting nurses’ opportunities to access flexible professional learning and continuously update the digital competencies required in rapidly evolving healthcare environments. For healthcare organizations, these barriers may reduce the effectiveness of continuing digital education strategies and hinder efforts to prepare the nursing workforce for ongoing digital transformation. Previous research has shown that nurses’ acceptance of M‐learning depends substantially on their perceptions of its usefulness and ease of use [2]. More broadly, recent evidence on digital nursing technologies indicates that insufficient training, low technological confidence, increased workload, and inadequate organizational support can hinder nurses’ adoption and effective use of digital technologies, whereas leadership support and targeted training facilitate successful implementation [5]. Therefore, identifying the technological, individual, and organizational factors that facilitate or hinder M‐learning adoption is important for nursing managers and healthcare organizations seeking to develop effective continuing digital education strategies.

Against this background, the successful implementation of M‐learning for CNE and professional development requires a comprehensive understanding of practicing nurses’ behavioral intention to adopt such technologies and the factors underlying these intentions. Behavioral intention refers to an individual’s intention or willingness to use a particular technology [6, 7]. In the present study, it represents nurses’ intention to use M‐learning CNE and professional development. In the existing literature, technology adoption behavior is explained through several well‐established theoretical frameworks. The most commonly used models include the Technology Acceptance Model (TAM), the Theory of Reasoned Action (TRA), and the Unified Theory of Acceptance and Use of Technology (UTAUT) [6, 8–10]. Although the UTAUT has demonstrated strong empirical power in explaining the general determinants of technology acceptance [6, 10, 11], it does not account for factors such as users’ attitudes and system quality, which may significantly influence adoption decisions. The DeLone and McLean Information System (IS) Success Model addresses these system characteristics through constructs such as information quality, which reflects the quality of information provided by a system, and system quality, which reflects the technical performance and usability of the system [12]. While certain studies have integrated the IS Success Model with the UTAUT or its extended version (UTAUT2), they have often examined these theoretical models separately in relation to behavioral intention [13–15]. Meanwhile, relatively few studies have incorporated the “quality” constructs of the IS Success Model into UTAUT2 as external variables for empirical validation. In the present study, attitude reflects nurses’ overall evaluation of using M‐learning, mobile self‐efficacy (MSE) represents their confidence in using mobile devices for learning, and technology anxiety reflects apprehension or discomfort associated with technology use.

Previous empirical studies have indicated that anxiety is one of the factors influencing users’ acceptance of technology‐enhanced learning [6, 16]. Although numerous studies have explored the use of various technologies in M‐learning, the specific determinants influencing its adoption remain underexplored.

The concept of self‐efficacy, which originates from Social Cognitive Theory (SCT) [17], emphasizes an individual’s belief in their ability to perform specific tasks. It has been recognized as a critical determinant of the acceptance and continued use of educational technologies [18]. With the widespread implementation of mobile devices and wireless networks, this concept has been extended to “MSE,” which has gained increasing academic attention and plays a key role in technology‐enhanced learning contexts [10, 19, 20].

Therefore, understanding the factors affecting nurses’ adoption and use of M‐learning is crucial for policymakers, hospital administrators, and technology developers. Despite the growing body of evidence, there remains a gap in integrating multiple theoretical frameworks to comprehensively understand the determinants of M‐learning adoption [16, 21–23].

To address this gap, this study integrates the UTAUT2 framework, the IS Success Model, and individual psychological factors such as attitude, MSE, and technology anxiety to develop a model for investigating the determinants of nurses’ behavioral intention to adopt M‐learning. The study posits that the proposed integrated model offers a comprehensive and explanatory theoretical framework for systematically examining key determinants of M‐learning adoption among nurses. Compared with a single‐theory perspective, this integrated model not only encompasses technological characteristics and system quality dimensions but also incorporates individual psychological traits and emotional responses, thereby providing a more holistic theoretical explanation.

Insights derived from this integrated model can assist nursing managers, healthcare administrators, and continuing education providers in developing targeted implementation strategies and support mechanisms. The goal is to enhance nurses’ acceptance and actual use of M‐learning, which in turn may improve professional competency development and clinical practice outcomes.

Within UTAUT2, performance expectancy refers to the perceived benefits of using a technology for improving performance, whereas effort expectancy refers to the perceived ease associated with its use [6, 7]. Based on the above, two research questions are proposed: (1) In the professional context of nurses in Taiwan, which key constructs within the UTAUT2 and attitude influence behavioral intention to adopt M‐learning? (2) How do nurses’ perceptions of information quality and system quality, as well as their MSE and technology anxiety, influence performance expectancy and effort expectancy within the UTAUT2 framework?

In addressing these research questions, this study makes three major theoretical and practical contributions: (1) By integrating the UTAUT2 and the IS Success Model, along with individual factors including MSE and technology anxiety, the study responds to calls by Venkatesh et al. [7] and DeLone and McLean [12] to explore additional determinants and extend model applications to new contexts; (2) It identifies information quality, system quality, MSE, and technology anxiety as significant drivers of performance expectancy and effort expectancy within the UTAUT2 framework, thereby supporting and extending prior research findings; and (3) It contributes to the development of effective CNE and professional development strategies for practicing nurses.

## 2. Literature Review

### 2.1. M‐Learning

M‐learning generally refers to learning activities supported through portable digital devices, such as smartphones and tablets, which enable learners to access educational resources and engage in learning across different times and locations [22]. It represents one of the key trends in the application of emerging technologies in education. Since its introduction in the early 2000s, M‐learning has gradually become a major research topic in educational technology. While the core concept was established at an early stage, its definition has continued to evolve and deepen alongside the rapid advancement of digital technologies and mobile devices.

The Policy Guidelines for M‐learning published by the United Nations Educational, Scientific, and Cultural Organization in 2013 highlighted that M‐learning is an important means of expanding and enriching educational opportunities and offers multiple advantages [24]. This perspective also aligns with the United Nations’ Sustainable Development Goal 4, which emphasizes the promotion of inclusive and equitable quality education [25].

In addition, previous studies have characterized M‐learning as a flexible mode of learning that can occur anytime and anywhere, without constraints of time, location, or equipment [15, 26, 27]. It also meets learners’ expectations for education that is immediate, appropriately scaled, and tailored to individual needs [28]. Some researchers conceptualize M‐learning as the use of handheld electronic devices (e.g., tablets, smartphones, and personal digital assistants) to access educational resources and engage in learning activities anytime and anywhere [22]. M‐learning is a learner‐centered instructional approach that provides highly personalized learning content and supports autonomous learning. Furthermore, it allows multiple learners to engage on the same digital platform through collaboration, discussion, and knowledge exchange, thereby enhancing learning interactions and outcomes [29].

Recent research has increasingly emphasized the role of digital learning technologies in CNE and CPD for practicing nurses. Tischendorf et al. [1] highlighted the growing need to develop digital competencies among nursing professionals through continuing education and training and emphasized the importance of systematically integrating digitalization into CPD. Mobile‐based approaches are particularly relevant in this context because they can provide flexible and accessible learning opportunities that accommodate nurses’ clinical workloads. Hilsmann and Dodson [30] further identified mobile microlearning as an emerging approach to nursing professional development, providing concise and flexible learning activities that may support nurses’ ongoing learning needs.

Beyond conventional M‐learning, emerging digital and intelligent educational technologies are also being explored for the CPD of practicing nurses. Nyiringango et al. [31] found that practicing nurses showed positive acceptance of virtual patients as an alternative approach to CPD, indicating the potential of such digital learning tools to support nurses’ ongoing professional learning in primary healthcare settings. Mayer et al. [32] further highlighted the growing use of extended‐reality technologies, including virtual, augmented, and mixed reality, in nursing professional development. More recently, Ariga et al. [33] found that telelearning development was positively associated with artificial intelligence (AI) readiness among clinical nurses, suggesting that supportive digital learning environments may contribute to nurses’ preparedness for emerging AI‐enabled healthcare technologies.

Against this background, M‐learning in the present study is defined as a flexible learning approach through which practicing nurses use handheld electronic devices, such as smartphones and tablets, to access CNE and professional development resources and participate in learning activities anytime and anywhere.

### 2.2. UTAUT

The UTAUT is one of the most important theoretical frameworks in the field of technology adoption and is primarily used to analyze users’ behavioral intention to adopt a given technology [34]. It was originally proposed by Venkatesh et al. [6] through the integration of eight established theoretical models related to technology use, cognition, and behavior, including the TRA, Theory of Planned Behavior, TAM, a model combining TAM and Theory of Planned Behavior, a model of personal computer utilization, SCT, the motivational model, and innovation diffusion theory [6, 10].

Empirical evidence indicates that the UTAUT framework can explain up to 70% of the variance in users’ behavioral intention to adopt technology, outperforming individual theoretical models [6, 34, 35]. It comprises four primary constructs influencing technology use intention and behavior, namely performance expectancy, effort expectancy, social influence, and facilitating conditions [6, 35]. Performance expectancy refers to the degree to which an individual believes that using a technology will improve task or performance outcomes, whereas effort expectancy refers to the perceived ease associated with using the technology. Social influence reflects the degree to which individuals perceive those important others believe they should use a particular technology, while facilitating conditions refer to the perceived availability of organizational and technical resources that support technology use [6, 35]. In the context of the present study, these constructs represent nurses’ perceptions of the professional benefits, ease of use, social expectations, and available support associated with M‐learning.

As an extension of the original UTAUT framework, the UTAUT2 incorporates additional constructs tailored to consumer contexts, such as habit, hedonic motivation, and price value [7]. Habit refers to the extent to which technology use becomes automatic as a result of prior learning and repeated experience [7]. In the present study, habit reflects the extent to which nurses’ use of mobile technologies for learning has become a routine or automatic behavior. Together with the four original constructs within the UTAUT, these additional constructs directly influence users’ behavioral intention to use technology [7]. Owing to its robustness and comprehensiveness, the UTAUT2 provides an appropriate theoretical framework for investigating the determinants of M‐learning adoption. More recently, Farooq et al. [36] extended UTAUT2 by incorporating personal innovativeness and referred to the resulting framework as UTAUT3. UTAUT3 retains the major constructs of UTAUT2, including performance expectancy, effort expectancy, social influence, facilitating conditions, hedonic motivation, price value, and habit, while adding personal innovativeness to account for individual differences in the willingness to adopt new technologies. Recent research has also applied and extended UTAUT3 in healthcare settings. For example, Ngusie et al. [37] applied an extended UTAUT3 model to examine health professionals’ intention to use electronic health record systems and found that personal innovativeness, performance expectancy, and attitude were significant predictors of behavioral intention.

Although UTAUT3 provides a broader extension of UTAUT2, the present study adopts UTAUT2 as the core theoretical framework and selects constructs that are particularly relevant to nurses’ professional M‐learning context. Hedonic motivation was excluded because M‐learning in this context primarily serves professional continuing education and competency development rather than entertainment‐oriented purposes. Price value was also excluded because M‐learning in the present professional context was not primarily conceptualized as a consumer purchase decision; therefore, the monetary cost–benefit trade‐off underlying price value was considered less salient.

In addition, moderating variables in the original UTAUT and UTAUT2 frameworks, such as gender, experience, and age, may not exert significant effects in certain applied contexts [13, 38]. Therefore, these variables were also excluded from the proposed model in this study. This simplification allows for the direct examination of relationships among constructs. UTAUT2 has been widely applied to technology‐supported learning contexts, although much of the earlier empirical evidence has been derived from student populations in academic settings. These studies provide general theoretical support for the explanatory value of UTAUT2 in educational technology adoption. For example, studies conducted in academic learning settings have demonstrated the applicability of UTAUT2 to blended learning, electronic learning platforms, mobile‐based educational applications, and M‐learning. Azizi et al. [34] found that UTAUT2 effectively explained students’ behavioral intention to adopt blended learning, whereas Prasetyo et al. [39] identified performance expectancy as a significant predictor of intention to use electronic learning platforms. Other studies have reported significant effects of performance expectancy, social influence, facilitating conditions, and habit on behavioral intention, although the significance and direction of these relationships have varied across educational contexts [14, 40].

More directly relevant to the present study, recent evidence among practicing nurses supports the applicability of technology‐acceptance factors to CPD and digital technology adoption in healthcare settings. Han et al. [41] examined nurses’ acceptance of digital healthcare technologies and identified performance expectancy and facilitating conditions as important factors associated with their intention to accept these technologies. Similarly, Nyiringango et al. [31] reported that PU and PEOU were positively associated with practicing nurses’ behavioral intention to adopt virtual patients for CPD. More broadly, Walzer et al. [5] identified insufficient training, low technological confidence, workload concerns, and organizational support as important factors influencing nurses’ implementation and adoption of digital nursing technologies. Collectively, these findings suggest that the adoption of digital technologies among practicing nurses is influenced by both individual perceptions of technology and the organizational conditions supporting its use. Behavioral intention is particularly relevant to the present study because it represents the primary outcome through which nurses’ acceptance of M‐learning is examined. Consistent with UTAUT and UTAUT2, behavioral intention plays a central role in explaining technology acceptance [6, 7]. Accordingly, examining the factors associated with behavioral intention provides a theoretical basis for understanding nurses’ acceptance of M‐learning for CNE and professional development.

Based on the above findings, this study posits that performance expectancy, effort expectancy, social influence, facilitating conditions, and habit within the UTAUT2 framework constitute key determinants of nurses’ behavioral intention to adopt M‐learning, and that effort expectancy directly affects performance expectancy. Accordingly, the following hypotheses are proposed:

• H1: Performance expectancy exhibits a positive effect on nurses’ behavioral intention to use M‐learning.
• H2: Effort expectancy exhibits a positive effect on nurses’ behavioral intention to use M‐learning.
• H3: Social influence exhibits a positive effect on nurses’ behavioral intention to use M‐learning.
• H4: Facilitating conditions exhibit a positive effect on nurses’ behavioral intention to use M‐learning.
• H5: Habit exhibits a positive effect on nurses’ behavioral intention to use M‐learning.
• H6: Effort expectancy exhibits a positive effect on nurses’ performance expectancy in using M‐learning.

Attitude refers to an individual’s overall favorable or unfavorable evaluation of using a particular technology [42, 43]. In the present study, attitude reflects the extent to which nurses evaluate the use of M‐learning positively or negatively for CNE and professional development. Prior empirical studies [35, 38, 44, 45] have found that performance expectancy and effort expectancy are important predictors of attitude within ISs research. However, the effect of effort expectancy on attitude remains inconclusive. For example, Šumak et al. [46] found that effort expectancy was not a significant predictor of attitude. Therefore, further investigation is warranted to clarify the influence of performance expectancy on attitude toward M‐learning, as well as whether effort expectancy promotes a more positive attitude among nurses. Accordingly, the following hypotheses are proposed:

• H7: Attitude exhibits a positive effect on nurses’ behavioral intention to use M‐learning.
• H8: Performance expectancy exhibits a positive effect on nurses’ attitude toward using M‐learning.
• H9: Effort expectancy exhibits a positive effect on nurses’ attitude toward using M‐learning.

### 2.3. IS Success Model

The IS Success Model, originally proposed by DeLone and McLean [12], is a well‐established and widely applied theoretical framework for evaluating the success of IS implementation and performance [8, 9]. The model comprises six key constructs of IS success, namely information quality, system quality, use, user satisfaction, individual impact, and organizational impact. In response to increasing user demands for service quality and evolving IS environments, DeLone and McLean [12] updated the IS Success Model by incorporating two additional constructs, i.e., “service quality” and “intention to use,” thereby better explaining user behavior in practice. Among various constructs, system quality, information quality, and service quality are regarded as critical factors influencing users’ acceptance of ISs [9]. Given the focus of the present study on the informational and technical characteristics of M‐learning that may shape nurses’ performance expectancy and effort expectancy, information quality and system quality were selected as the two quality dimensions incorporated into the proposed model. Service quality was not included because service‐related support was outside the scope of the present study. Information quality refers to the perceived quality of the information produced or provided by an IS, including characteristics such as accuracy, relevance, completeness, and timeliness. System quality refers to the perceived technical performance of an IS, including its reliability, usability, accessibility, and functionality [12]. In the context of M‐learning, information quality therefore reflects nurses’ perceptions of the accuracy, relevance, completeness, and usefulness of the learning content provided, whereas system quality reflects their perceptions of the reliability, usability, accessibility, and technical performance of the M‐learning system. In the present study, system quality and information quality from the IS Success Model proposed by DeLone and McLean [12] were incorporated as predictors of performance expectancy and effort expectancy. Specifically, this study examines how these quality dimensions influence nurses’ performance expectancy and effort expectancy toward M‐learning.

The IS Success Model by DeLone and McLean [12] has been frequently applied in studies investigating key success factors of various online learning systems, such as M‐learning [23], blended electronic learning systems [8], and electronic learning systems [9]. Although some of the earlier evidence in technology‐supported learning was derived from student populations, these studies provide theoretical support for the relationships between system‐related quality factors and users’ perceptions of learning technologies. In a study utilizing the IS Success Model to explore factors associated with the adoption of M‐learning among university students, Al‐Nassar [23] found that information quality, system quality, and service quality significantly influenced students’ perceptions of M‐learning. Similarly, in a study integrating the IS Success Model with the TAM to examine nurses’ intention to use blended electronic learning, Tsai et al. [8] found that information quality and system quality significantly influenced both PU and PEOU, whereas service quality significantly influenced PEOU but not PU. Cheng [9] further demonstrated that system quality, information quality, and user‐interface design quality affected PU, PEOU, and perceived enjoyment, while service quality significantly influenced PU and PEOU.

More recently, Salama et al. [47] proposed the Unified MOOC Utilization Model (UMUM) by integrating the DeLone and McLean IS Success Model with the task–technology fit framework to explain students’ reuse and overall utilization of MOOCs. Their findings showed that quality‐related factors, including system quality and information quality, positively influenced fit‐related factors, which subsequently contributed to technology utilization. Their findings further highlighted the importance of system quality and information quality in technology‐supported learning utilization. Consistent with this perspective, Liu et al. [48] applied an integrated IS Success Model–TAM framework to M‐learning and demonstrated the relevance of system‐related quality factors to PU and PEOU. Given the conceptual similarity between performance expectancy and PU and between effort expectancy and PEOU, respectively, these findings provide a theoretical basis for examining whether information quality and system quality also influence performance expectancy and effort expectancy in M‐learning contexts. However, relatively few studies have directly examined these relationships within a UTAUT‐based framework, particularly among practicing nurses engaged in CNE and professional development. Therefore, the present study incorporates information quality and system quality as external predictors of performance expectancy and effort expectancy to examine nurses’ adoption of M‐learning. Accordingly, the following hypotheses are proposed:

• H10: Information quality exhibits a positive effect on performance expectancy.
• H11: Information quality exhibits a positive effect on effort expectancy.
• H12: System quality exhibits a positive effect on performance expectancy.
• H13: System quality exhibits a positive effect on effort expectancy.

### 2.4. MSE

Self‐efficacy is regarded as one of the key factors influencing the acceptance of educational systems [15]. Originating from SCT [17], self‐efficacy refers to an individual’s belief in their ability to successfully perform specific tasks, achieve designated goals, or overcome difficulties. Importantly, self‐efficacy does not refer to the actual skills or abilities possessed by an individual, but rather their subjective perception and judgment of those abilities [10, 17]. According to Bandura, individuals with high self‐efficacy are more likely to perceive difficult tasks as challenges to be overcome rather than obstacles to be avoided [17]. With the integration of multiple technologies, such as telecommunication and wireless networks, into mobile devices since the late 2000s, new usage patterns have emerged along with associated risks. Consequently, as an extension of self‐efficacy in mobile technology contexts, MSE has received increasing academic attention. Existing literature suggests that MSE plays a pivotal role in the application of mobile devices in educational support contexts and serves as an important theoretical basis for understanding users’ attitudes and usage patterns toward these devices [10, 19, 20, 49].

Nikou and Economides [19] define MSE as an individual’s perception of their ability to use mobile devices to accomplish specific tasks, where higher levels of MSE indicate greater confidence in using mobile devices to achieve intended objectives [19, 20]. In the present study, MSE refers specifically to nurses’ confidence in their ability to use mobile devices effectively to access and participate in CNE and professional development activities. Furthermore, Nikou and Economides [19] found that MSE directly influences PEOU. Venkatesh [50] also identified self‐efficacy as a predictor of PEOU. Similarly, Shao et al. [51] found that higher levels of AI self‐efficacy enhance both PEOU and PU of AI. Recent evidence from practicing nurses further highlights the relevance of self‐efficacy‐related beliefs in adapting to emerging intelligent technologies. Atalla et al. [52], in a study of nurses working in critical care units, found that positive AI‐related attitudes were significantly associated with creative self‐efficacy and clinical reasoning competency. Although creative self‐efficacy is conceptually distinct from MSE, these findings suggest that efficacy‐related beliefs remain relevant to practicing nurses’ adaptation to emerging technologies. The authors also emphasized targeted training and CPD as important strategies for preparing nurses for AI‐integrated clinical environments. Nonetheless, empirical evidence regarding the effects of MSE on performance expectancy and effort expectancy remains limited. Given the conceptual correspondence between performance expectancy and PU and between effort expectancy and PEOU, the present study proposes that nurses with higher MSE are more likely to perceive M‐learning as useful for enhancing their professional performance and as requiring less effort to use. Accordingly, the following hypotheses are proposed:

• H14: MSE exhibits a positive effect on attitude.
• H15: MSE exhibits a positive effect on performance expectancy.
• H16: MSE exhibits a positive effect on effort expectancy.

### 2.5. Technology Anxiety

Perceived anxiety refers to the concern and fear experienced by an individual regarding potential negative consequences associated with the introduction of new technologies or methods [53]. Technology anxiety can be understood as the fear or concern individuals experience when considering or beginning to use unfamiliar computer technologies [54]. It reflects users’ psychological state regarding their ability and willingness to use technology‐related tools [55] and involves anxiety or fear arising from the prospect of using new technologies [6]. In the present study, technology anxiety specifically refers to nurses’ anxiety or concern associated with using mobile technologies for learning. Existing research has established that anxiety significantly influences variables related to technology adoption. Earlier evidence from higher education settings showed that investigating the effects of information and communication technology anxiety on higher education, Mac Callum et al. [16] found that information and communication technology anxiety significantly affected both teachers’ and students’ intentions to use technology, with higher anxiety associated with lower usage intentions. Additionally, among the student cohort, information and communication technology anxiety negatively affects PU and PEOU. Notably, Gunasinghe and Nanayakkara [56] reported that technology anxiety positively influences performance expectancy and effort expectancy, while Thanigan et al. [53] found that perceived anxiety affects effort expectancy but not performance expectancy. More directly relevant to practicing nurses, recent evidence further underscores the relevance of anxiety in the adoption of emerging intelligent technologies. Ma and Wang [57] demonstrated substantial heterogeneity in AI anxiety among newly recruited nurses and found that AI training experience, AI proficiency, attitudes toward AI, and self‐efficacy were significantly associated with different anxiety profiles. Although AI anxiety is context‐specific and conceptually distinct from the broader construct of technology anxiety examined in the present study, these findings suggest that anxiety remains an important psychological factor in nurses’ responses to emerging intelligent technologies. Taken together, prior research indicates that technology‐related anxiety is an important factor in technology adoption; however, empirical findings regarding its effects on performance expectancy and effort expectancy remain inconsistent. Given the conceptual correspondence between performance expectancy and PU and between effort expectancy and PEOU, higher levels of technology anxiety may reduce users’ perceptions of the usefulness and ease of using a technology [16]. Therefore, further investigation is warranted to examine whether technology anxiety negatively influences performance expectancy and effort expectancy in the context of M‐learning among practicing nurses. Accordingly, the following hypotheses are proposed:

• H17: Technology anxiety exhibits a negative effect on performance expectancy.
• H18: Technology anxiety exhibits a negative effect on effort expectancy.

**FIGURE 1:**
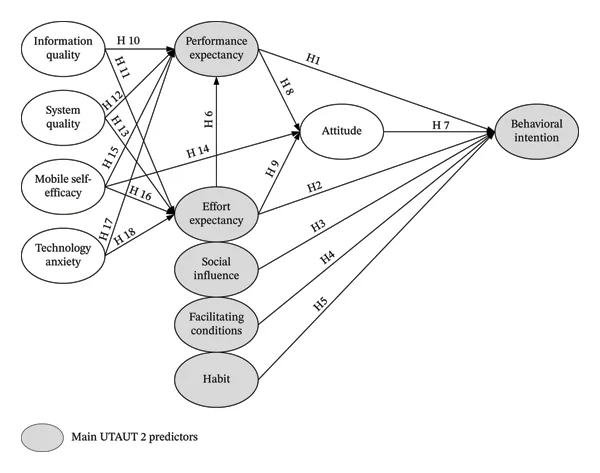
Research framework.

Based on the above findings, in this study, the UTAUT2 framework proposed by Venkatesh et al. [7] was integrated with the “information quality” and “system quality” constructs of the IS Success Model proposed by DeLone and McLean [12] and individual psychological factors such as MSE and technology anxiety to develop a new theoretical model for examining the determinants of nurses’ behavioral intention to adopt M‐learning. Subsequently, through empirical data collection and analysis, the performance of the proposed model was verified using a structural equation modeling approach. Research variables incorporated in the proposed model include (1) seven exogenous variables, i.e., information quality, system quality, MSE, technology anxiety, social influence, facilitating conditions, and habit and (2) four endogenous variables, i.e., performance expectancy, effort expectancy, attitude, and behavioral intention. Within the proposed framework, behavioral intention serves as the primary outcome construct representing nurses’ intention to adopt M‐learning. Performance expectancy, effort expectancy, and attitude are examined as key determinants that are directly or indirectly associated with behavioral intention. Information quality and system quality are modeled as predictors of performance expectancy and effort expectancy, while MSE is examined as a predictor of attitude, performance expectancy, and effort expectancy. Technology anxiety is examined as a predictor of performance expectancy and effort expectancy, whereas social influence, facilitating conditions, and habit are specified as direct determinants of behavioral intention. The research framework is illustrated in Figure 1.

## 3. Research Methodology

### 3.1. Measurement Instrument

All variables were measured using a five‐point Likert scale, with scores ranging from 1 (“strongly disagree”) to 5 (“strongly agree”). The proposed model comprises 11 constructs, all of which were assessed using validated instruments from prior studies, with appropriate modifications made to align with the specific context and objectives of this study.

A total of 51 measurement items across 11 constructs were used to examine the relationships between the proposed factors and behavioral intention. The questionnaire items are presented in Appendix. Specifically, the primary constructs of the UTAUT2 framework, i.e., performance expectancy, effort expectancy, social influence, facilitating conditions, habit, and behavioral intention, were measured using 29 items developed by Venkatesh et al. [6] and Venkatesh et al. [7] and validated in previous studies [10, 14, 38, 46]. Attitude was measured using four items adapted from prior research [38, 46]. Meanwhile, information quality (four items) and system quality (five items) were assessed using nine items developed by Tsai et al. [8], Cheng [9], and DeLone and McLean [12]. Finally, MSE was measured using five items developed by Chao [10], Nikou and Economides [19], and Ozturk et al. [20], while technology anxiety was assessed using four items adapted from Mac Callum and Jeffrey [16] and Meuter et al. [55].

### 3.2. Sampling and Data Collection

Using a nonprobability convenience sampling method, nurses from a regional teaching hospital in Yunlin, Taiwan, were recruited as research subjects. Data were collected via a self‐administered questionnaire. After the questionnaire was drafted, five experienced experts in information technology, nursing education, and questionnaire design were invited to review and refine the instrument to ensure content validity and to minimize any semantic discrepancies [58]. Prior to the formal survey, a pilot study involving 85 participants was conducted to validate the questionnaire design. Data collected during the pilot phase were not included in the final analysis. The results indicated that all constructs achieved a Cronbach’s *α* above 0.7 (ranging from 0.832 to 0.946) [59], reflecting good internal consistency and reliability of the measurement items.

### 3.3. Sample Characteristics

Data were collected from 173 nurses employed at a regional teaching hospital in Yunlin, Taiwan. Prior to questionnaire distribution, the purpose and content of the survey were explained to participants to ensure informed and voluntary participation. Upon completion, participants were instructed to deposit their questionnaires into a sealed collection box, which was subsequently retrieved by the research team to minimize potential response bias and safeguard respondent anonymity. Nurses on unpaid leave during the data collection period were excluded from the study. This sample size satisfies the recommendation by Hair et al. [60] that the minimum sample size for model and hypothesis testing should be five to 10 times the number of factors (which equates to a minimum of 110 participants for the 11 factors included in the study). A total of 173 valid responses were ultimately included for model estimation and hypothesis testing. SPSS Version 24.0 was used for data screening and descriptive statistical analysis, while SmartPLS Version 4.0 was employed for structural equation modeling to test the proposed model and hypotheses.

Among the 173 respondents, the mean age was 35.12 years (SD = 7.69). Regarding nursing clinical ladder level, participants were classified according to Taiwan’s hospital‐based clinical ladder system, which stratifies nurses into five progressive levels (N0–N4) based on clinical competency, years of experience, and professional performance, with higher levels reflecting greater clinical autonomy and expertise. In this study, the majority of participants were classified as N2 (*n* = 87, 50.2%), indicating that most respondents possessed intermediate‐level clinical competency and moderate professional experience. Regarding educational background, most participants held a university (college) degree (*n* = 156, 90.2%). With respect to tenure at the current hospital, the largest subgroup had worked for more than nine years (*n* = 51, 29.5%), suggesting that a substantial proportion of respondents had accumulated considerable institutional and clinical experience. Detailed demographic characteristics are presented in Table 1.

**TABLE 1:** Demographic characteristics of nurse participants (*n* = 173).

Participant characteristic	*n*	Percentage
Nursing level		
N1	43	24.9
N2	87	50.2
N3	33	19.1
N4	5	2.9
Head Nurse	5	2.9
Education level		
Nursing school	10	5.8
University (college)	156	90.2
Graduate degree or above	7	4.0
Tenure at the current hospital		
< 1 year	16	9.2
1–< 3 years	34	19.7
3–< 5 years	31	17.9
5–< 7 years	16	9.2
7–< 9 years	25	14.5
≥ 9 years	51	29.5

### 3.4. Ethical Considerations

This study was approved by the Institutional Review Board of Changhua Christian Hospital, Changhua Christian Medical Foundation (approval number: 201227). The research process strictly adhered to the principles of the Helsinki Declaration and complied with standard ethical guidelines for human subject research. All participants were required to read an informed consent statement prior to completing the questionnaire, which emphasized voluntary participation and assured data confidentiality. Completion of the questionnaire following review of the consent statement was considered to constitute informed consent. Participants were informed that no personally identifiable information was collected, and all data were anonymized prior to analysis to ensure the protection of privacy.

## 4. Results

Following the recommendations of Hair et al. [60] for structural equation modeling, a two‐stage analytical procedure was adopted. In the first stage, confirmatory factor analysis was conducted to evaluate the reliability and validity of the measurement model. In the second stage, structural equation modeling was performed to assess the structural model. Data analysis was conducted using SmartPLS version 4.0 (with bootstrapping of 5000 resamples and a significance level of 0.05). The results are described as follows.

### 4.1. Common Method Variance (CMV)

In studies utilizing a cross‐sectional survey design, the presence of CMV may potentially introduce bias. In this study, three procedural and statistical remedies were performed to mitigate the impact of CMV. First, respondents were assured of anonymity. Additionally, questionnaire items measuring independent and dependent variables were carefully arranged to avoid close proximity [61]. Second, Harman’s single‐factor test [62] was conducted to identify CMV. The results showed that a single factor accounted for 37.71% of the variance, which is below the 50% threshold recommended by Podsakoff et al. [61]. Third, Bagozzi et al. [63] suggested that correlation coefficients exceeding 0.9 would indicate the presence of CMV. In this study, the highest correlation was observed between attitude and behavioral intention (*r* = 0.877; Table 2), which remains below the 0.9 threshold. Collectively, these findings indicate the absence of significant CMV within the study.

**TABLE 2:** HTMT results.

	1.	2.	3.	4.	5.	6.	7.	8.	9.	10.	11.
1. Information quality	**0.902**	0.918	0.527	0.286	0.553	0.407	0.537	0.556	0.360	0.511	0.538
2. System quality	0.856	**0.885**	0.460	0.228	0.554	0.547	0.562	0.668	0.347	0.414	0.439
3. MSE	0.491	0.430	**0.901**	0.268	0.172	0.174	0.258	0.476	0.133	0.492	0.509
4. Technology anxiety	−0.264	−0.213	−0.250	**0.915**	0.274	0.053	0.111	0.173	0.117	0.145	0.229
5. Performance expectancy	0.514	0.516	0.115	−0.255	**0.876**	0.434	0.585	0.520	0.303	0.411	0.423
6. Effort expectancy	0.380	0.506	0.164	0.000	0.398	**0.873**	0.604	0.586	0.204	0.321	0.265
7. Social influence	0.503	0.529	0.244	−0.055	0.549	0.569	**0.922**	0.732	0.290	0.580	0.609
8. Facilitating conditions	0.522	0.620	0.446	−0.161	0.482	0.543	0.689	**0.887**	0.392	0.570	0.637
9. Habit	0.336	0.321	0.083	0.055	0.279	0.185	0.273	0.393	**0.868**	0.259	0.336
10. Attitude	0.473	0.386	0.463	−0.136	0.386	0.298	0.551	0.533	0.240	**0.923**	0.935
11. Behavioral intention	0.497	0.407	0.476	−0.212	0.393	0.244	0.574	0.593	0.310	0.877	**0.913**

*Note:* The bold diagonal values represent the square roots of AVE. Values above the diagonal indicate HTMT, while values below the diagonal represent interconstruct correlation coefficients. HTMT: Heterotrait–Monotrait ratio.

Abbreviation: MSE, mobile self‐efficacy.

### 4.2. Measurement Model

Before testing the hypotheses in the structural model, the measurement model was first evaluated in terms of reliability and convergent validity. The results reflected adequate reliability (Table 3), as the Cronbach’s *α* of all constructs exceeded the threshold of 0.7 recommended by Hair et al. [59]. Regarding convergent validity, all composite reliability values exceeded the recommended threshold of 0.7 [64]. Furthermore, factor loadings for all measurement items were above 0.7 and reached statistical significance (*p* < 0.05) [59], indicating that items accurately represent their respective latent constructs and demonstrate strong robustness. Lastly, the average variance extracted for all constructs exceeded the acceptable threshold of 0.5 recommended by Fornell and Larcker [64], confirming adequate convergent validity. Detailed reliability and validity assessment results are presented in Table 3.

**TABLE 3:** CR and AVE of all constructs.

Construct	No. of items	Factor loading	Cronbach’s *α*	rho_*α*	CR	AVE
1. Information quality	4	0.867–0.923	0.924	0.929	0.946	0.815
2. System quality	5	0.834–0.907	0.931	0.938	0.948	0.784
3. MSE	5	0.760–0.954	0.941	0.955	0.955	0.812
4. Technology anxiety	4	0.882–0.954	0.935	0.941	0.954	0.838
5. Performance expectancy	5	0.812–0.917	0.924	0.933	0.943	0.767
6. Effort expectancy	5	0.833–0.895	0.922	0.928	0.941	0.762
7. Social influence	5	0.898–0.940	0.956	0.961	0.966	0.850
8. Facilitating conditions	5	0.828–0.925	0.932	0.933	0.949	0.788
9. Habit	5	0.806–0.927	0.920	0.965	0.938	0.753
10. Attitude	4	0.885–0.960	0.942	0.942	0.958	0.852
11. Behavioral intention	4	0.876–0.934	0.933	0.936	0.952	0.833

*Note:* Cronbach’s alpha: α, rho_a: Dijkstra–Henseler’s rho.

Abbreviations: AVE, average variance extracted; CR, composite reliability.

The results of the discriminant validity analysis are presented in Table 2 and were conducted to assess whether the included constructs are empirically distinct [64]. In this study, discriminant validity was evaluated using the Heterotrait–Monotrait ratio. The findings indicate that, except for the pairs of information quality and system quality and attitude and behavioral intention, all Heterotrait–Monotrait ratio values (above the diagonal) were below the recommended threshold of 0.85 [65]. Furthermore, all interconstruct correlation coefficients (below the diagonal) were lower than the square roots of the average variance extracted (diagonal values), indicating satisfactory discriminant validity. Overall, these results provide strong evidence supporting the discriminant validity of the included constructs.

### 4.3. Structural Model

Based on the recommendations of prior studies, the structural model was evaluated using three indices, i.e., standardized root mean square residual (SRMR), goodness‐of‐fit, predictive relevance (*Q* ^2^), effect sizes (*f* ^2^), and coefficient of determination [59, 66, 67]. According to the analysis, the SRMR value (0.066) remained within the acceptable range of below 0.08–0.10 advocated by Hair et al. [68], while the GoF value (0.603) exceeded the 0.36 benchmark reported in the literature [66, 67], jointly confirming a satisfactory model fit. The *R* ^2^ estimates further indicated that the model explained 81.3%, 34.4%, 37.4%, and 28.1% of the variance in behavioral intention, attitude, performance expectancy, and effort expectancy, respectively. To complement this variance‐based assessment, the relative magnitude of each predictor’s influence was evaluated using Cohen’s *f* ^2^ criterion, with 0.02, 0.15, and 0.35 denoting small, medium, and large effects [69]. Finally, predictive relevance was verified via the *Q* ^2^ statistic, where values exceeding zero denote adequate predictive capability for the corresponding construct [70]; as all constructs in this study returned positive *Q* ^2^ values (0.234–0.431), the model can be regarded as both well‐fitting and predictively robust.

**FIGURE 2:**
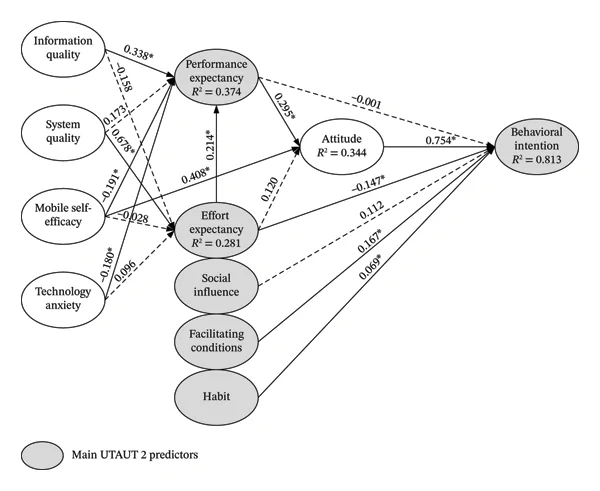
Path diagram of the proposed model. Note: ^∗^ *p* < 0.05.

Analysis results using the structural model (Table 4 and Figure 2) indicate that behavioral intention is significantly and positively influenced by facilitating conditions (H4: *β* = 0.167, *t* = 2.579, *f* ^2^ = 0.063), habit (H5: *β* = 0.069, *t* = 2.565, *f* ^2^ = 0.022), and attitude (H7: *β* = 0.754, *t* = 13.825, *f* ^2^ = 1.938), with attitude being the most pronounced factor. In contrast, effort expectancy (H2: *β* = −0.147, *t* = 2.624, *f* ^2^ = 0.073) exerts a significant negative effect on behavioral intention. Therefore, H4, H5, and H7 are supported. Meanwhile, since performance expectancy (H1: *β* = −0.001, *t* = 0.021, *f* ^2^ = 0.000) and social influence (H3: *β* = 0.112, *t* = 1.301, *f* ^2^ = 0.027) show no significant effects on behavioral intention, H1, H2, and H3 are not supported. Effort expectancy (H6: *β* = 0.214, *t* = 2.442, *f* ^2^ = 0.053) has been shown to have a significant positive effect on performance expectancy, thereby supporting H6.

**TABLE 4:** Path coefficients and *t* values of the structural model.

Hypothesis	Path	Coefficient	*t* value	Test result
H1	Performance expectancy ⟶ Behavioral intention	−0.001	0.021	Rejected
H2	Effort expectancy ⟶ Behavioral intention	−0.147^∗^	2.624	Rejected
H3	Social influence ⟶ Behavioral intention	0.112	1.301	Rejected
H4	Facilitating conditions ⟶ Behavioral intention	0.167^∗^	2.579	Supported
H5	Habit ⟶ Behavioral intention	0.069^∗^	2.565	Supported
H6	Effort expectancy ⟶ Performance expectancy	0.214^∗^	2.442	Supported
H7	Attitude ⟶ Behavioral intention	0.754^∗^	13.825	Supported
H8	Performance expectancy ⟶ Attitude	0.295^∗^	4.045	Supported
H9	Effort expectancy ⟶ Attitude	0.120	1.466	Rejected
H10	Information quality ⟶ Performance expectancy	0.338^∗^	2.106	Supported
H11	Information quality ⟶ Effort expectancy	−0.158	1.138	Rejected
H12	System quality ⟶ Performance expectancy	0.173	1.083	Rejected
H13	System quality ⟶ Effort expectancy	0.678^∗^	5.172	Supported
H14	MSE ⟶ Attitude	0.408^∗^	6.898	Supported
H15	MSE ⟶ Performance expectancy	−0.191^∗^	2.007	Rejected
H16	MSE ⟶ Effort expectancy	−0.028	0.309	Rejected
H17	Technology anxiety ⟶ Performance expectancy	−0.180^∗^	2.405	Supported
H18	Technology anxiety ⟶ Effort expectancy	0.096	1.096	Rejected

^∗^ *p* < 0.05.

Attitude is significantly and positively influenced by performance expectancy (H8: *β* = 0.295, *t* = 4.045, *f* ^2^ = 0.111) and MSE (H14: *β* = 0.408, *t* = 6.898, *f* ^2^ = 0.245), with MSE being the stronger predictor. Therefore, H8 and H14 are supported. Conversely, effort expectancy (H9: *β* = 0.120, *t* = 1.466, *f* ^2^ = 0.018) does not significantly affect attitude, leading to the rejection of H9. While information quality (H10: *β* = 0.338, *t* = 2.106, *f* ^2^ = 0.045) exhibits a significant positive effect on performance expectancy, its correlation with effort expectancy is not significant (H11: *β* = −0.158, *t* = 1.138, *f* ^2^ = 0.009). Consequently, H10 is supported, while H11 is rejected. System quality (H13: *β* = 0.678, *t* = 5.172, *f* ^2^ = 0.171) significantly and positively affects effort expectancy, but not performance expectancy (H12: *β* = 0.173, *t* = 1.083, *f* ^2^ = 0.011). Therefore, H13 is supported, whereas H12 is rejected. MSE exerts a significant negative effect on performance expectancy (H15: *β* = −0.191, *t* = 2.007, *f* ^2^ = 0.043). However, its effect on effort expectancy fails to reach statistical significance (H16: *β* = −0.028, *t* = 0.309, *f* ^2^ = 0.001). As a result, both H15 and H16 are rejected. Finally, technology anxiety has a significant negative effect on performance expectancy (H17: *β* = −0.180, *t* = 2.405, *f* ^2^ = 0.046), but not on effort expectancy (H18: *β* = 0.096, *t* = 1.096, *f* ^2^ = 0.012). Therefore, H17 is supported, while H18 is not.

## 5. Discussion

As an extension of the original UTAUT framework, UTAUT2 is capable of explaining nurses’ behavioral intention to adopt M‐learning. However, it does not account for external variables related to quality and individual psychological factors. The objective of this study was to investigate the determinants of nurses’ behavioral intention to adopt M‐learning. For this purpose, a theoretical framework was developed by integrating the IS Success Model into the UTAUT2 framework and incorporating MSE and technology anxiety as external factors to provide a reliable explanation of nurses’ behavioral intention to adopt M‐learning. The performance of the proposed model was subsequently evaluated using the data collected from 173 nurses at a regional teaching hospital in Yunlin, Taiwan. The findings indicate that most of the constructs included significantly influence nurses’ behavioral intention to use M‐learning and that the proposed model is both theoretically sound and explanatory in identifying the determinants of behavioral intention to adopt M‐learning.

The results suggest that when nurses perceive M‐learning as beneficial for their work and learning, they develop a more positive attitude toward it. This finding is consistent with previous studies [35, 38, 44, 45], which found that performance expectancy has a positive effect on nurses’ attitudes toward using M‐learning. These findings indicate that healthcare administrators can enhance nurses’ attitudes by developing M‐learning systems that clearly demonstrate their learning benefits.

In contrast, performance expectancy does not significantly influence behavioral intention, suggesting that it is not a key driver of usage intention. Although prior studies have identified performance expectancy as a major determinant of behavioral intention [6, 7, 39], some research suggests that its influence may be limited in the context of new ISs or emerging technologies [35]. In this study, the lack of support regarding the effects of performance expectancy may indicate that, in certain contexts (e.g., hospitals), respondents’ usage intentions toward M‐learning systems are compromised by organizational requirements or limited understanding of new ISs.

The findings further reveal a negative effect of effort expectancy on behavioral intention, such that nurses who perceived the M‐learning system as more complex or time‐consuming reported lower intention to use it. This diverges from prior UTAUT‐based studies in both healthcare and educational settings, which have generally reported positive or nonsignificant associations between effort expectancy and behavioral intention [6, 7, 34]. Unlike students or general users in educational contexts, nurses operate under substantial time pressure and cognitive load from direct patient care duties; consequently, additional effort required to navigate the system may be perceived as a competing demand on already constrained resources. This effect may be further amplified by nurses’ concern about privacy [71], including fears of cyberattacks or personal data breaches, given that M‐learning systems often involve sensitive professional and patient‐related information. These findings suggest that the effort expectancy–behavioral intention relationship is more context‐dependent than previously assumed, warranting greater attention to healthcare‐specific boundary conditions in future research. In addition, the effect of effort expectancy on attitude is not significant, suggesting that effort expectancy is not a significant predictor of attitude. This finding is somewhat unexpected, as previous studies have identified effort expectancy as an important predictor of attitude in ISs research [35, 38, 44, 45]. This may be attributed to the fact that M‐learning has become deeply embedded in nurses’ work and learning contexts, and modern mobile device interfaces are increasingly intuitive, thereby reducing operational barriers. As a result, effort expectancy may no longer play a critical role in shaping attitude.

The findings also demonstrate that facilitating conditions, habit, and attitude show are significant positive determinants of behavioral intention, with attitude being the most significant factor. This finding supports previous research suggesting that attitude is a key determinant of behavioral intention [44, 45]. Specifically, nurses are more likely to adopt M‐learning when they hold a positive attitude toward it and have convenient access to resources.

In addition to the UTAUT2, two constructs of the IS Success Model, i.e., information quality and system quality, were also incorporated in the proposed model. The results indicate that information quality is the most important predictor of performance expectancy, while system quality is the key determinant of effort expectancy, consistent with prior findings [8, 9]. Nurses who place greater importance on information quality are more likely to perceive M‐learning as beneficial for their work and study when they consider its content to be rich and up to date. Therefore, efforts should be made to develop learner‐centered courses and provide timely, high‐quality information to enhance learners’ engagement in M‐learning [9]. Nurses who are more sensitive to system quality tend to perceive M‐learning as easier to use when the system demonstrates strong interactivity, usability, and visual presentation. To improve the usability of M‐learning interfaces, healthcare administrators and system developers should carefully evaluate the alignment between system functionality and nurses’ needs, thereby enhancing behavioral intention. Nonetheless, since most nurses already possess basic digital literacy and are familiar with mobile device operations, they may be able to complete tasks even when system design or stability is suboptimal, which may explain why the observed effect of system quality on effort expectancy is insignificant in this study.

The results further highlight the critical role of individual psychological factors in nurses’ use of M‐learning, particularly the effects of MSE and technology anxiety on performance expectancy and effort expectancy. Both MSE and technology anxiety exert negative effects on performance expectancy, while neither exhibits a significant effect on effort expectancy. These findings differ from prior research [19, 50, 51, 53, 56], suggesting that the relationships may vary across contexts and populations. In this study, when nurses lack confidence in their ability to use mobile technologies or experience anxiety and discomfort when interacting with technology, their belief in the usefulness of M‐learning for improving work and learning performance is diminished. Furthermore, as nurses generally possess basic digital literacy and experience with mobile devices, they are already familiar with and adaptable to the operations of M‐learning systems. Under such conditions, even if individuals experience psychological discomfort or lack confidence, these emotional factors may no longer significantly influence their perceptions of system usability. This may explain why neither MSE nor technology anxiety significantly affects effort expectancy.

Beyond the constructs examined in this study, the rapid advancement of generative AI and intelligent learning technologies is poised to reshape the landscape of M‐learning in nursing education, warranting further discussion. In clinical practice, the adoption of generative AI has the potential to enhance the efficiency of nursing workflows by producing personalized case scenarios, simulating patient interactions, and delivering instant clinical guidance, thereby reinforcing nurses’ perceived performance expectancy beyond what conventional static ISs can offer. The integration of intelligent tutoring systems (ITS) into M‐learning platforms may further address the effort‐related concerns identified in this study; by dynamically adapting content difficulty and pacing to individual learners′ competency levels, ITS‐enabled platforms could reduce the cognitive burden associated with navigating complex system interfaces, potentially reversing the negative effect of effort expectancy observed among nurses in this study. Adaptive M‐learning systems, which leverage AI algorithms to continuously adjust content sequencing based on real‐time learner performance, may likewise offer more responsive clinical education, particularly valuable given the substantial heterogeneity in clinical experience and competency levels (e.g., N0–N4) observed among the nursing workforce. Building on this, the development of personalized learning pathways, tailored to each nurse’s clinical ladder level and prior knowledge, may further enhance engagement and long‐term retention of clinical knowledge. Finally, AI‐assisted clinical education holds considerable promise for bridging the gap between theoretical M‐learning content and hands‐on clinical practice: beyond delivering up‐to‐date clinical knowledge, AI‐assisted systems can help identify individual nurses’ specific learning gaps and competency deficits, thereby enabling precision learning that targets each learner’s unique developmental needs rather than adopting a uniform, one‐size‐fits‐all instructional approach. Given that individual psychological factors such as MSE and technology anxiety were found to significantly shape nurses’ perceptions in this study, future research should further examine how nurses’ readiness to engage with AI‐driven educational tools, and their trust in AI‐generated clinical guidance, may serve as additional psychological factors influencing the adoption of next‐generation M‐learning systems in nursing education.

### 5.1. Theoretical Implications

This study makes several contributions to existing theoretical frameworks and literature. First, by integrating the UTAUT2 framework [7], the information quality and system quality constructs within the IS Success Model [12], and individual psychological factors (i.e., MSE and technology anxiety), a novel theoretical framework was developed that examines nurses’ behavioral intention to adopt M‐learning. The findings deepen the understanding of nurses’ use of M‐learning in work and learning contexts and provide the first empirical validation of the integrated model. Additionally, they offer further support for both the UTAUT2 and the IS Success Model and respond to prior calls for testing theoretical models across diverse contexts. Second, according to the results, the hypotheses regarding performance expectancy and social influence affecting behavioral intention are not supported, nor is the hypothesis that effort expectancy influences attitude or that system quality and information quality influence performance expectancy and effort expectancy. These observations are inconsistent with prior findings and open new avenues for future research focusing on nurses’ characteristics and their work and learning contexts.

Third, additional individual psychological factors (i.e., MSE and technology anxiety) are included in the discussion. According to the results, the hypotheses that MSE or technology anxiety influences effort expectancy are not supported, suggesting that psychological factors may primarily affect nurses’ perceptions of the benefits of M‐learning (e.g., performance expectancy) rather than their PEOU (e.g., effort expectancy).

Overall, this study not only extends the application of the UTAUT2 and the IS Success Model in healthcare and M‐learning contexts but also offers theoretical insights into nurses’ psychological characteristics, professional learning environments, and technology adoption behavior. These findings lay a foundation for future cross‐cultural and interdisciplinary research.

### 5.2. Practical Implications

Beyond the constructs examined in this study, the rapid advancement of generative AI and intelligent learning technologies is poised to reshape the landscape of M‐learning in nursing education, warranting further discussion. In clinical practice, the adoption of generative AI has the potential to enhance the efficiency of nursing workflows by producing personalized case scenarios, simulating patient interactions, and delivering instant clinical guidance, thereby reinforcing nurses’ perceived performance expectancy beyond what conventional static ISs can offer. The integration of ITS into M‐learning platforms may further address the effort‐related concerns identified in this study; by dynamically adapting content difficulty and pacing to individual learners’ competency levels, ITS‐enabled platforms could reduce the cognitive burden associated with navigating complex system interfaces, potentially reversing the negative effect of effort expectancy observed among nurses in this study. Adaptive M‐learning systems, which leverage AI algorithms to continuously adjust content sequencing based on real‐time learner performance, may likewise offer more responsive clinical education, particularly valuable given the substantial heterogeneity in clinical experience and competency levels (e.g., N0–N4) observed among the nursing workforce. Building on this, the development of personalized learning pathways, tailored to each nurse’s clinical ladder level and prior knowledge, may further enhance engagement and long‐term retention of clinical knowledge. Finally, AI‐assisted clinical education holds considerable promise for bridging the gap between theoretical M‐learning content and hands‐on clinical practice: beyond delivering up‐to‐date clinical knowledge, AI‐assisted systems can help identify individual nurses’ specific learning gaps and competency deficits, thereby enabling precision learning that targets each learner’s unique developmental needs rather than adopting a uniform, one‐size‐fits‐all instructional approach. Given that individual psychological factors such as MSE and technology anxiety were found to significantly shape nurses’ perceptions in this study, future research should further examine how nurses’ readiness to engage with AI‐driven educational tools, and their trust in AI‐generated clinical guidance may serve as additional psychological factors influencing behavioral intention to adopt next‐generation M‐learning systems in nursing education. For nursing educators, these technologies may support the design of competency‐based and personalized learning activities that better address nurses’ varying clinical experience and learning needs. By strengthening nurses’ clinical knowledge, decision‐making, and competency development, mobile and AI‐assisted learning may also contribute indirectly to the quality and safety of patient care.

## 6. Limitations and Future Outlook

This study has several limitations that warrant consideration and offer directions for future research. First, the data were collected from a single country and region, namely, Yunlin, Taiwan, which constrains the generalizability of the findings. Given the substantial cross‐national variation in cultural values, digital infrastructure, and technology‐related regulatory environments, the determinants identified in this study may not fully translate to other cultural or institutional contexts. Future research is encouraged to replicate and validate the proposed model across multiple countries and regions and to conduct cross‐country comparative analyses to examine whether the hypothesized relationships hold consistently or vary systematically across cultural settings. Relatedly, employing multigroup analysis (MGA) to formally test measurement invariance and compare path coefficients across demographic or contextual subgroups (e.g., age cohorts, educational levels, urban versus rural populations, or countries with differing digital infrastructure maturity) would provide more rigorous evidence regarding the boundary conditions and cross‐cultural robustness of the proposed model.

Second, this study adopted a cross‐sectional research design, with data collected within a relatively short time frame. As user perceptions, technology familiarity, and behavioral patterns may evolve over time, the cross‐sectional nature of the data limits the ability to draw causal inferences and precludes the observation of how relationships among constructs may shift as users gain experience with M‐learning platforms. Future studies employing a longitudinal design, with data collected at multiple time points, are recommended to more rigorously establish causal ordering among constructs and to capture the dynamic, evolving nature of user adoption behavior—for instance, examining how initial adoption intentions translate into sustained usage or discontinuance over time. Third, this study examined M‐learning adoption exclusively from the end‐user perspective. Future research could enrich this analytical scope by incorporating the perceptions, attitudes, and readiness of other key stakeholders, such as healthcare providers, instructors, or institutional administrators, thereby offering a more holistic and multistakeholder understanding of M‐learning implementation.

Fourth, this study was primarily grounded in established theoretical frameworks, namely, UTAUT2 and the IS Success Model (encompassing information quality and system quality). Nevertheless, behavioral intention toward M‐learning is likely shaped by a broader constellation of factors not fully captured by these frameworks. Future research should consider incorporating variables such as digital literacy, AI readiness, organizational support, and learning engagement. Integrating these constructs, whether as direct predictors, moderators, or mediators, alongside alternative theoretical lenses such as the stimulus–organism–response (S‐O‐R) paradigm or the technology readiness model, would enable future studies to more comprehensively identify the multifaceted determinants of M‐learning adoption and to better reflect the evolving technological landscape in which learners increasingly interact with AI‐driven educational tools.

## Funding

This study was supported by the Changhua Christian Hospital under grant number 109‐CCH‐IRP‐202.

## Conflicts of Interest

The authors declare no conflicts of interest.

## Data Availability

The data used to support the findings of this study are available from the corresponding authors upon reasonable request.

## Appendix A

**Table A1:** Questionnaire items.

Construct	Item
1. Information quality	The mobile learning system allows me to easily access up‐to‐date learning information.
	The information provided by the mobile learning system is helpful for my nursing work and learning.
	The classification and indexing of information in the mobile learning system are clear and easy to navigate.
	The content and information presented in the mobile learning system are accurate and reliable.

2. System quality	The mobile learning system provides comprehensive functions that meet my needs.
	The mobile learning system operates stably, with minimal crashes or delays.
	The mobile learning system performs efficiently when downloading data or processing information.
	The operational steps of the mobile learning system are clear and easy to understand.
	The interface design and visual presentation of the mobile learning system are attractive.

3. MSE	I can successfully use the mobile learning system if someone else has demonstrated how to operate it.
	I feel confident in my ability to operate the mobile learning system independently.
	I can easily use various relevant information and support tools to operate the mobile learning system.
	I can operate the mobile learning system independently by referring to online instructions or user manuals.
	When encountering difficulties, I can still use the system as long as assistance is available.

4. Technology anxiety	I feel resistant or fearful when using new technological products or systems.
	When using new technological services, I often worry that improper operation may cause system malfunction.
	I tend to avoid using new technologies because I find them difficult to operate.
	I try to avoid using new technologies because I am afraid that I will not know how to fix errors.

5. Performance expectancy	The mobile learning system is helpful for my daily nursing work and clinical learning.
	The mobile learning system improves my efficiency in completing learning goals or work tasks.
	The mobile learning system helps me complete work and learning tasks more efficiently.
	The mobile learning system allows me to flexibly access required health education information without constraints of time and location.
	The mobile learning system helps me carry out clinical health education activities more effectively.

6. Effort expectancy	Learning how to operate the mobile learning system is easy for me.
	The interface and interaction processes of the mobile learning system are clear and easy to understand.
	I can quickly become proficient in using various functions of the mobile learning system.
	I do not need to spend excessive time or effort to learn how to use the mobile learning system.
	Overall, I find the mobile learning system easy to learn and use.

7. Social influence	People who influence my behavior think that I should use the mobile learning system.
	People who influence my work (e.g., supervisors and colleagues) think that I should use the mobile learning system.
	My family and friends support my decision to use the mobile learning system.
	My nursing peers or healthcare colleagues support my use of the mobile learning system.
	The fact that many nursing colleagues around me use the mobile learning system encourages me to use it as well.

8. Facilitating conditions	I possess the ability to resolve problems encountered when using the mobile learning system.
	I can obtain assistance from others when I encounter difficulties using the mobile learning system.
	The mobile learning system is compatible with other systems I currently use.
	I possess the necessary knowledge to use the mobile learning system.
	I have the resources required to use the mobile learning system.

9. Habit	Using the mobile learning system has become a habit for me.
	I naturally use the mobile learning system when I need to learn or search for information.
	I prioritize using the mobile learning system in my learning or work.
	The mobile learning system has become an important tool in my daily life.
	For me, using the mobile learning system is a natural activity.

10. Attitude	Using the mobile learning system is a wise choice.
	Using the mobile learning system for continuing education or to assist with work is an excellent approach.
	I welcome and support the integration of the mobile learning system into my daily learning or nursing work.
	Overall, I hold a favorable attitude toward using the mobile learning system.

11. Behavioral intention	In the future, I intend to continue (or begin) using the mobile learning system.
	Whenever possible, I will frequently use the mobile learning system to support my learning or work.
	In the future, when I have continuing education or health education needs, I will prioritize using the mobile learning system.
	I will recommend the mobile learning system to other nursing colleagues.
